# SAR Observation and Modeling of Gap Winds in the Prince William Sound of Alaska

**DOI:** 10.3390/s8084894

**Published:** 2008-08-22

**Authors:** Haibo Liu, Peter Q Olsson, Karl Volz

**Affiliations:** 1 Alaska Experimental Forecast Facility, University of Alaska Anchorage, 2811 Merrill Field Dr, Anchorage, Alaska 99508, USA; 2 Alaska State Climate Center, Environmental and Natural Resources Institute, University of Alaska Anchorage, Alaska 99508, USA

**Keywords:** SAR, model, gap wind, barrier, Alaska

## Abstract

Alaska's Prince William Sound (PWS) is a unique locale tending to have strong gap winds, especially in the winter season. To characterize and understand these strong surface winds, which have great impacts on the local marine and aviation activities, the surface wind retrieval from the Synthetic Aperture Radar data (SAR-wind) is combined with a numerical mesoscale model. Helped with the SAR-wind observations, the mesoscale model is used to study cases of strong winds and relatively weak winds to depict the nature of these winds, including the area of extent and possible causes of the wind regimes. The gap winds from the Wells Passage and the Valdez Arm are the most dominant gap winds in PWS. Though the Valdez Arm is north-south trending and Wells Passage is east-west oriented, gap winds often develop simultaneously in these two places when a low pressure system is present in the Northern Gulf of Alaska. These two gap winds often converge at the center of PWS and extend further out of the Sound through the Hinchinbrook Entrance. The pressure gradients imposed over these areas are the main driving forces for these gap winds. Additionally, the drainage from the upper stream glaciers and the blocking effect of the banks of the Valdez Arm probably play an important role in enhancing the gap wind.

## 1. Introduction

The Prince William Sound (PWS) is located in the northern Gulf of Alaska(GOA). While relatively exposed to open ocean on its southern boundary, to the north, east and west, PWS is embayed by a rugged, elevated, and highly glaciated mountain barrier. In the cold season this barrier tends to separate the cold, dry interior continental air mass from the relatively warm, moist maritime airmass in the northern GOA. Frequently occurring low pressure systems in combination with the high terrain act to induce a variety of strong off-shore and along-shore local winds [12, 25]. In the cold season, the interior of Alaska often is dominated by high pressure near the surface, and the PWS relatively low pressure. Consequently, pressure gradients are maintained around the PWS and gap flows are common in major gaps and fjords in PWS.

A further unique characteristic of Prince William Sound is that it contains Port Valdez along its north boundary. Port Valdez is the southern terminus of the Trans Alaskan Pipeline System (TAPS). Hence, almost all the crude oil extracted in Alaska transits the full N-S extent of PWS in supertankers en route to ports of call beyond the GOA. The potential perils of such a method of oil transport were partially realized in 1989, with the infamous accidental grounding of the tanker *Exxon Valdez* and attendant massive oil spill. While the *Exxon Valdez* incident did not ultimately result from adverse weather conditions, it brought into sharp focus the potential consequences of navigating the PWS with its highly localized adverse weather conditions [23].

Gap flows are common and gusty [12] in PWS. The strength of these gap winds is strongly affected by the imposed pressure gradients. Strong pressure gradients often generate strong gap winds. Many previous studies on gap winds in the Northern GOA have been conducted over major gaps and consisted largely of in-situ aircraft observations. For an example, Macklin et al. [12] documented airplane observations of gap flows in Copper River and Resurrection Bay; Macklin et al. [13] also reported an airplane observation of a case involving gap flow in the lower Cook Inlet. These studies rely on in-flight observations limited in areal extent and are further constrained by the limitation of the observing instruments and instrument platforms. For example, an instrumented aircraft must fly a finite height above ground, inherently missing observations in close proximity to the surface.

Numerical weather prediction (NWP) models and satellite observations have also been combined to study gap winds. Streenburgh et al. [24] studied a gap wind in Tehuantepec of Mexico using MM5 and satellite images. Pan and Smith [18] studied gap winds in the Alaska Aleutians using a shallow water model and space-borne Synthetic Aperature Radar (SAR) images. Sandvikv and Furevik [21] studied high latitude gap winds at Spitsbergen, Norway. Winstead et al. [25] used SAR image and mesoscale models to better understand barrier jets in Northern Gulf of Alaska. Liu et al. [9] reported a climatology of simulated gap winds in Cook Inlet and Shelikof Strait of Alaska.

Space-borne Synthetic Aperture Radar (SAR) observed normalized radar cross section (NRCS) depends upon scattering from surface roughness elements within the beam footprint. The NRCS from water represents the roughness of the water body surface which is a non-linear function of near-surface wind stress. With a prior estimate of the wind direction, wind speed can be inverted from the NRCS [25]. Comparisons with buoys, models and scatterometer estimates have demonstrated that the accuracy of wind speed estimated from SAR is comparable to those of operational scatterometers when the proper wind direction is used in the NRCS inversion [15, 22]. At the Johns Hopkins University (JHU) Applied Physics Laboratory (APL), the Alaska SAR Demonstration Project has been operating for almost a decade. High resolution “snapshots” of the near-surface wind speed field have been processed using the Navy Operational Global Atmospheric Prediction System (NOGAPS) winds as a first guess along with the NRCS intensity observation [7] and compared well with buoy observations in GOA [6].

These SAR images are found online at http://fermi.jhuapl.edu/sar/stormwatch/web_wind

This site archives a vast number of the near-surface wind field images associated with a wide variety of mesoscale and synoptic-scale atmospheric phenomena. Using this rich archive, several studies have investigated boundary layer convection [1], gap flows [9, 10], barrier jets [11], polar lows [22], and synoptic-scale fronts [26].

This study uses SAR wind imagery from the JHU APL archive and a high-resolution (1 km in horizontal grid spacing) mesoscale model to document gap winds for gaps of small lateral dimension in PWS. Section 2 describes two cases of gap winds captured by SAR in PWS. Section 3 presents the mesoscale modeling of these two events. Section 4 discusses the simulated characteristics of the gap wind, and is followed by the conclusions in Section 5.

## 2. SAR observed Gap winds in Valdez and Wells Passage

**Figure 1.**
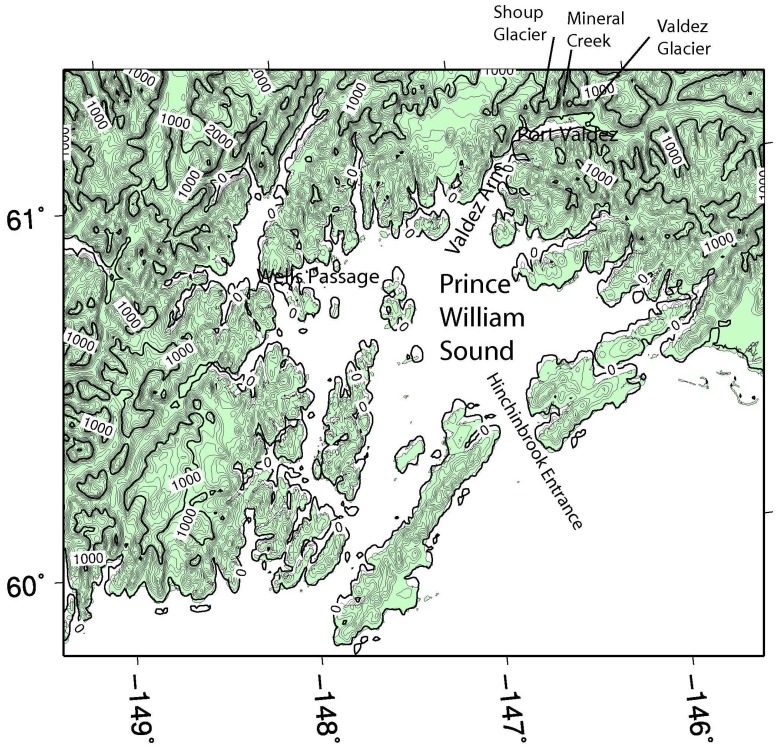
Map showing Prince William Sound. The landscape features referred to in the text are labeled. The terrain height contour spacing is 100m.

The Valdez Arm is a north-south trending gap located in the northern coast of PWS while the Wells Passage is a broader east-west oriented sea level gap (Fig.1) among the elevated coast lands and islands in western PWS. The Wells Passage is the extension of the relatively low elevation areas connecting the Cook Inlet and PWS. The Valdez Arm is an opening in the mountains of the north-central coast of the PWS, winds frequently blow from these channels— sometimes simultaneously— into the center of PWS.

**Figure 2.**
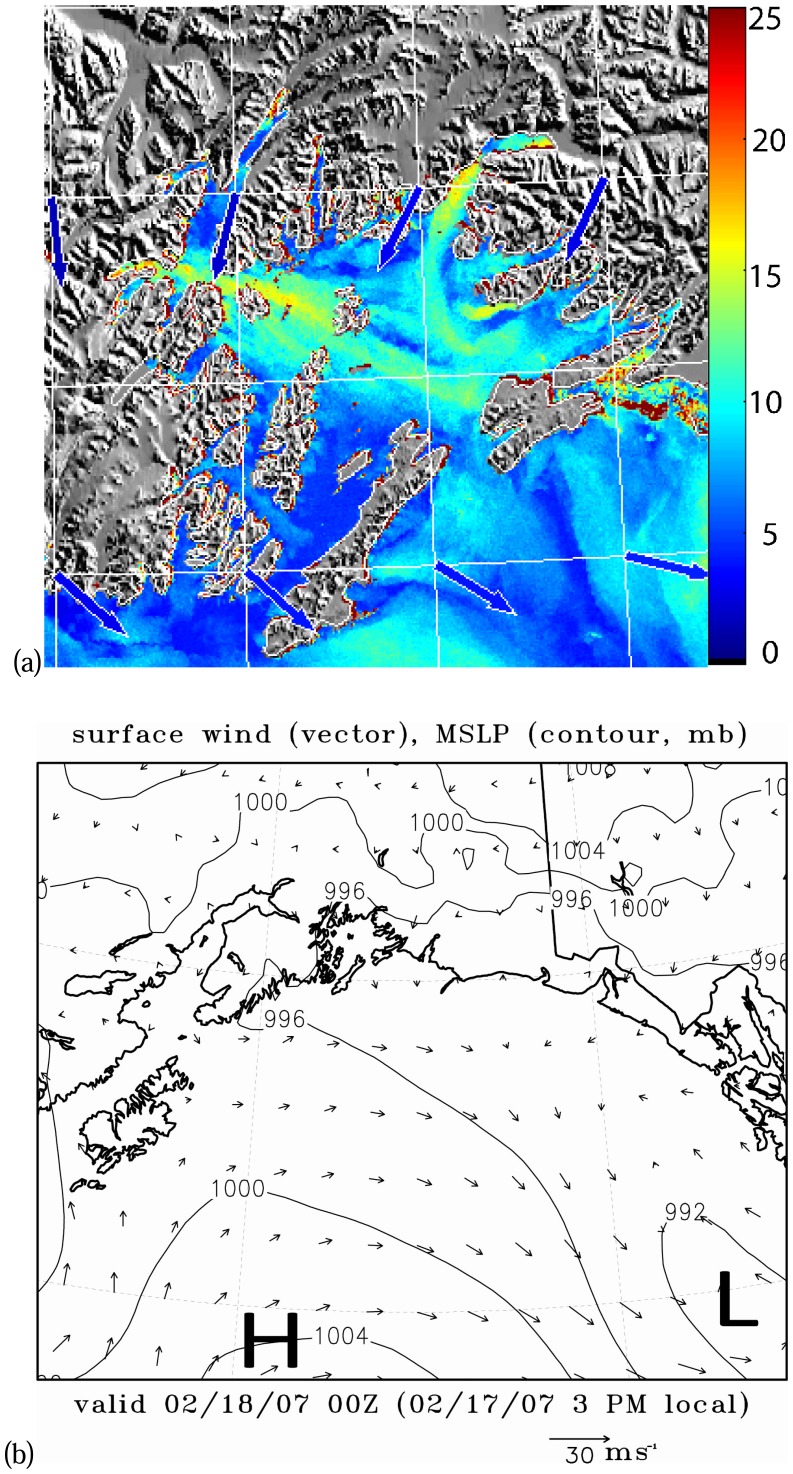
Wind event on 18 Feb 2007. (a) SAR-wind image. Wind speed is in ms^-1^. The arrows show the wind directions from the NOGAPS model. (b) Surface analysis closest in time to the SAR image.

Figure 2a is a SAR wind image which captures a weak gap wind event in the Valdez Arm and the Wells Passage respectively at 3:29 UTC 18 Feb 2007. The arrows in the image represent NOGAPS wind directions used in the computation of the SAR wind speeds [14, 26]. This image shows that the wind speed in the Valdez Arm and the Wells Passage are in the 12 to 17 ms^-1^ range. Relatively weak westerly winds dominate the PWS suppressing the northerly channeled wind exiting the Valdez Arm. The boundaries between the westerly flow and the north-easterly flow are clearly shown as a narrow zone of weak wind on the image. (Note the SAR wind image is a bit off in position compared to the land mask; the image should be moved a few pixels northeastwards. This slight offset in navigation induced thin red areas around coastlines in the image which can be falsely interpreted as strong winds.) Figure 2b shows the 0Z surface analysis which corresponds closely in time to this SAR image. The low system in the Northern Gulf of Alaska is moving southeastwards (not shown) away from PWS causing the pressure gradient in the PWS to gradually relax. At the point in time considered here, the pressure gradients induced by the high pressure in the Interior and the low pressure in PWS are weak. Consequently, the gap winds are weak in the PWS.

**Figure 3.**
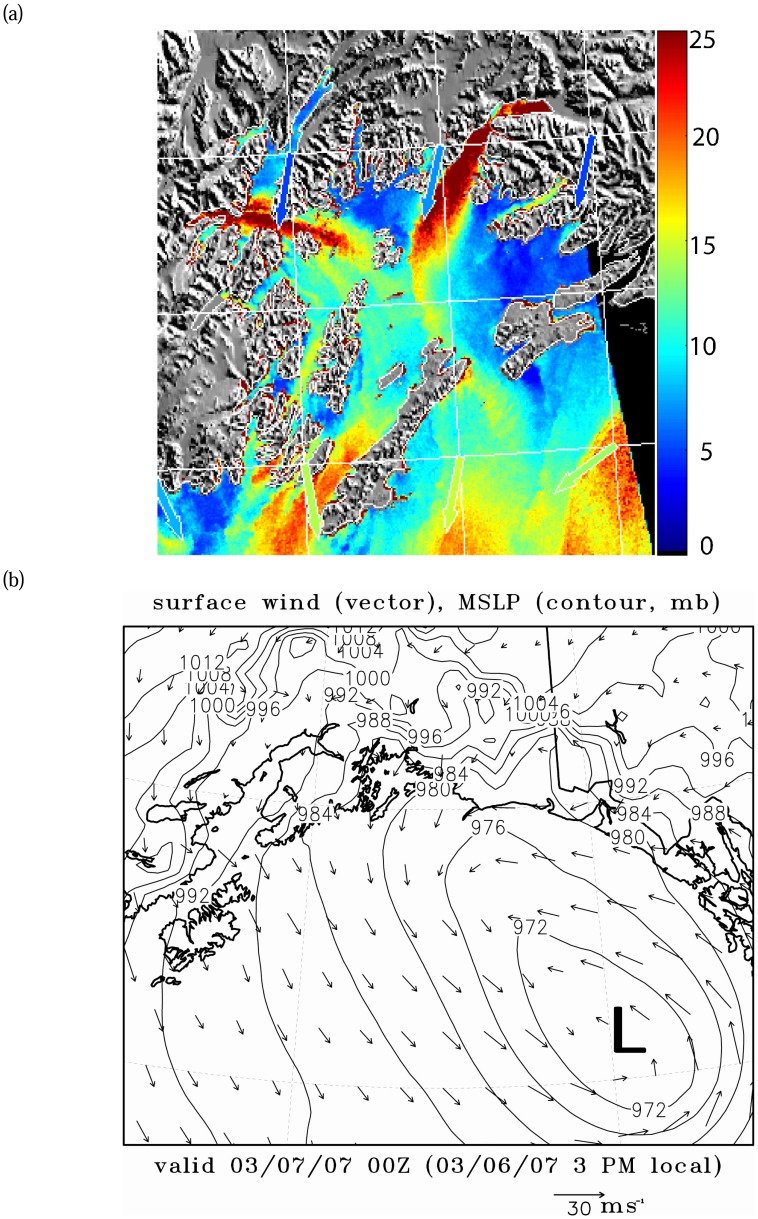
Wind event on 7 Mar 2007. (a) SAR-wind image, (b) surface analysis.

The Figure 3a SAR wind image shows a strong northeasterly gap wind of approximately 25 ms^-1^ in the Valdez Arm and a westerly strong gap wind of approximately the same strength from the Wells Passage. These two perpendicularly aligned jets meet near the center of the PWS. The combined wind jet further extends southeastwards out the PWS through the Hinchinbrook Entrance with a weaker speed of 15 ms^-1^. The extension of the Wells Passage jet turns right as the result of pushing from the Valdez jet and the right-turning nature of a gap wind [24]. Note, as in Figure 2, there is also a slight offset of the image relative to the land mask which results in some narrow red areas along the coastlines. The NOGAPS winds used in the SAR wind retrieval inversion are northerly in the PWS and its vicinity. Clearly, the coarse NOGAPS wind does not resolve the local winds well in the western PWS, which may cause significant wind speed errors [6, 25]. (How the deficiency of NOGAPS wind direction affects the accuracy of the wind retrieval is beyond the scope of this study.) Apparently, the Wells Passage winds are very strong in this case. However, the SAR-wind retrieval for the Valdes Arm has proper wind direction for both cases, which are our focus in this study. Figure 3b shows the surface synoptic pattern. A low system in the Gulf of Alaska is moving further northwest towards PWS. The high pressure in the Interior and the falling pressure in the northern Gulf form strong pressure gradients around the PWS which in turn induce strong gap winds in the Valdez Arm and the Wells Passage.

The above SAR images capture a weak wind event and a strong wind event respectively; and the surface pressure charts show there are subtle yet significant differences between these two to reveal the details of processes governing these two events, we use a numerical mesoscale atmospheric model to perform high resolution simulations. The following section introduces the model and discusses the simulation results.

## 3. RAMS simulation

### 3.1 Model description

**Figure 4.**
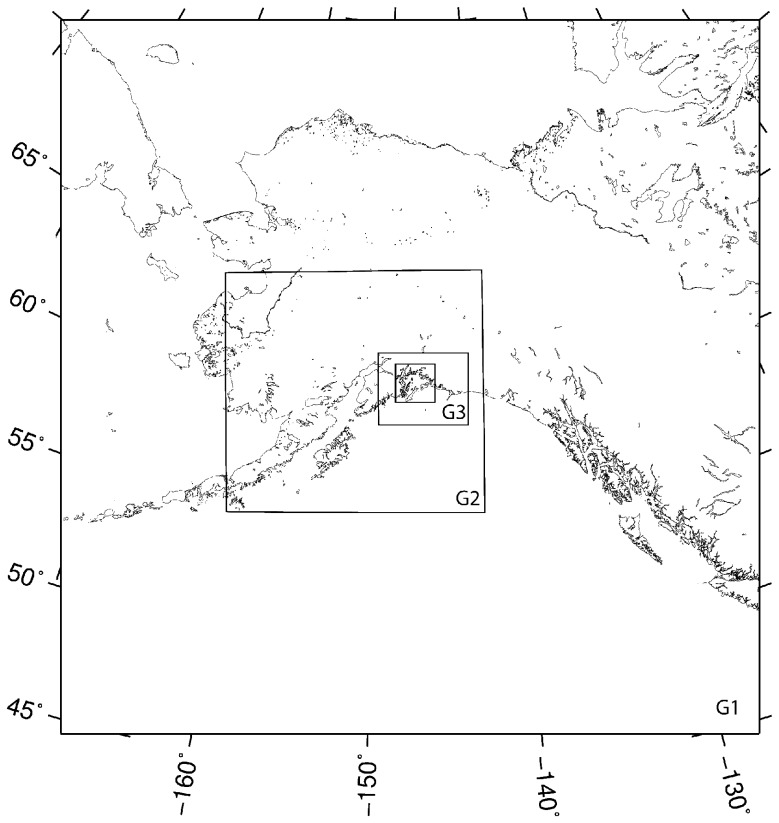
A schematic of model grid setting for this study. Grid-1 has 50 by 50 grid points with grid spacing of 64 km, Grid-2 70 by 58 grid points with spacing of 16 km, Grid-3 102 by 82 grid points with spacing of 4 km and the finest grid (Grid-4) 178 by 174 grid points with spacing of 1 km.

**Figure 5.**
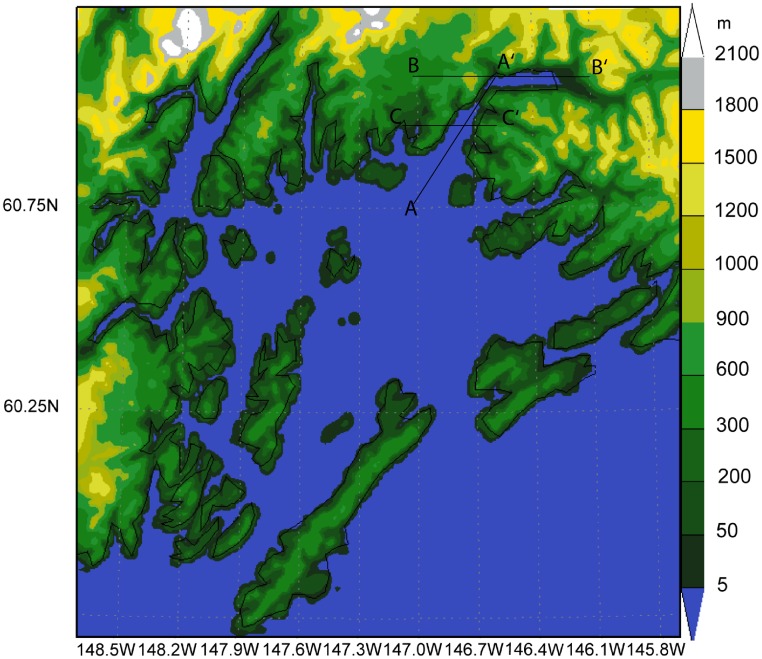
Model grid-4 resolved topography of PWS. All the area below 5 m is represented as ocean in this figure. The labeled lines represent the locations of cross sections referred in later sections.

We used the Regional Atmospheric Modeling System (RAMS) as our numerical tool in this study. Developed at Colorado State University and Mission Research Corporation, RAMS is a multipurpose numerical simulation system [20, 3]. It is a nonhydrostatic primitive-equation, finite-difference model that includes parameterizations for mixed phase microphysics, radiation, and planetary surface processes. RAMS provides a wide range of options that allow it to be tailored for a broad spectrum of applications. It contains a variety of structures and features ranging from nonhydrostatic codes, resolution ranging from less than a meter to hundreds kilometers, domains from a few kilometers to the entire globe, and a suite of physical options [20]. RAMS is well suited to simulate mesoscale phenomena including gap events [8, 4, 16, 9, 10]. We configured RAMS to run on four two-way nested grids. Grid-1 has 50 by 50 grids points with grid spacing of 64 km, sufficient to capture the synoptic-scale storm events. Grid-2 has 70 by 58 grid points with spacing of 16 km. Grid-3 has 102 by 82 grid points with spacing of 4 km and grid 4, the grid of interest in this study has 178 by 174 grid points with spacing of 1 km (Figure 4). Grid-4 covers the entire area of PWS and is fine enough to simulate the details of the gap flow. Figure 5 shows the grid-4 resolved topography of the PWS. The mountain ridges and gaps among the mountains are well represented. Vertically, all the four domains have the same 36 levels. The vertical grid spacing starts at 50 m at the surface and stretches by a factor of 1.15 for each successive level above the surface, to a maximum separation of 1200 m. To keep both the mountain heights and the deep valley effects on circulations in each domain, the “reflected envelope orography” scheme [20] is used. The digital elevation model (DEM) used here is at 30-second resolution. The coarse grid time step is 60 seconds and the integration results are written every hour. The sea surface temperature is from the weekly updated National Oceanic and Atmospheric Administration (NOAA) Optimal Interpolation sea surface temperature accessible at http://www.nodc.noaa.dsdt/oisst. The land use, vegetation information, and topography data are from the standard RAMS data sets. The simulations were carried out for 24 hours with initial and lateral forcing data from the National Centers for Environmental Prediction (NCEP) Eta model.

### 3.2 Simulation of the 18 Feb 2007 case

**Figure 6.**
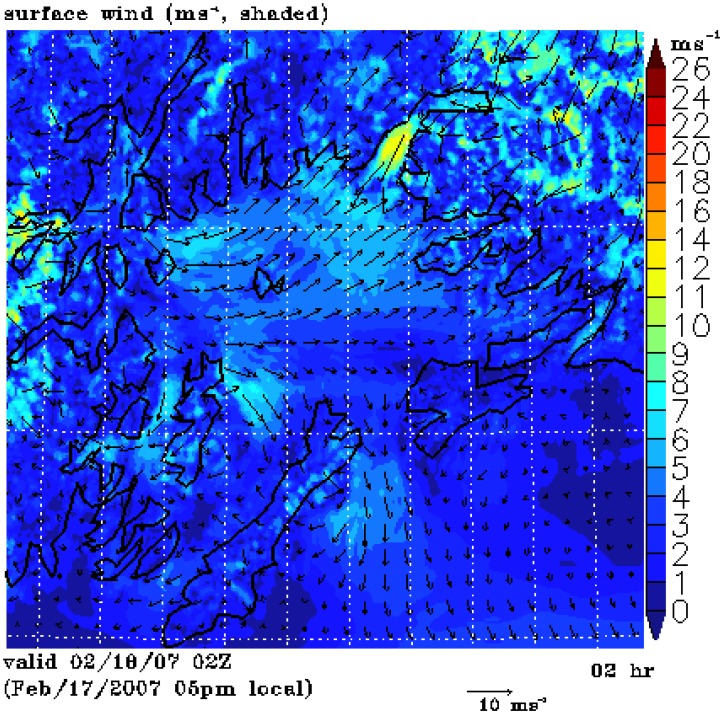
Surface wind for case A at 2 UTC 18 Feb 2007.

Figure 6 shows the surface winds for the 18 Feb 2007 case (Case A). The RAMS simulation has a speed of 12-14 ms^-1^ in the Valdez Arm and a weaker flow, 7-9 ms^-1^, in the Wells Passage. Note the model time is about 1.5 hours earlier than the corresponding SAR wind image. The reason for choosing the simulation of this time is that the RAMS wind most closely resembles the SAR wind (Figure 2a). Although the model gives a weaker wind along the Wells Passage, the wind has a pattern similar to that which can be inferred on the corresponding SAR image. The westerly winds dominate the PWS and the Valdez Arm gap wind is confined in the Arm. The convergence zone (with calm horizontal winds) between the westerly wind and the northerly Valdez Arm jet at the exit of the Arm is clearly visible. Winds in the north and east of the Port Valdez (reference Figure 1 for location) are directed into the Arm and have a high speed, indicating the likely source of the Valdez Arm jet are converging winds from the vicinity of Port Valdez.

**Figure 7.**
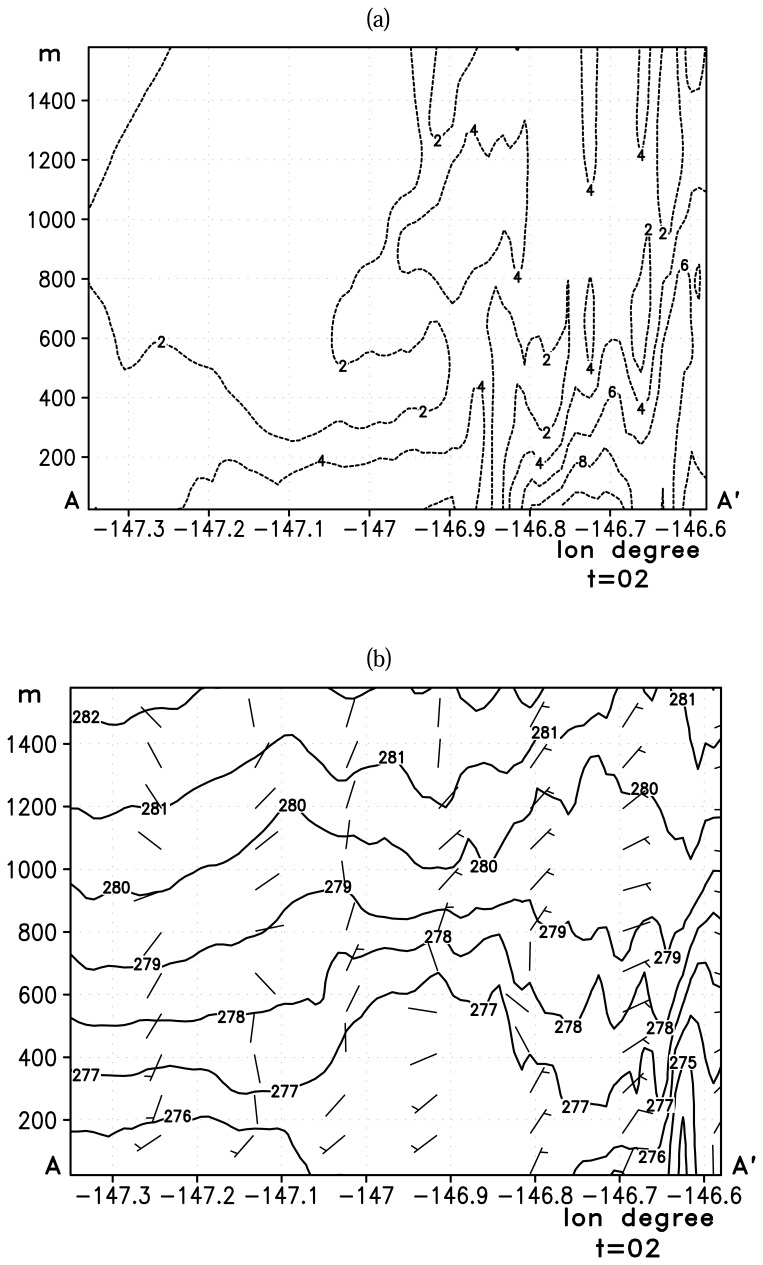
Vertical cross-section along the line AA′ (Figure 5) depicting (a): horizontal wind speed (ms^-1^) and (b): potential temperature (K) along the center of the jet for the Case A. The contour interval is 2 ms^-1^ for the wind speed and 1 K for the potential temperature. The x axis is longitude in degrees and y axis is height above the sea level in meters. A full bar of a wind barb represents 5 ms^-1^.

Figure 7a shows the vertical cross-section of wind (location AA′ in Fig.5) up to 1500 m above the sea surface along the center of Valdez Arm. The Valdez Arm is located between 146.9W and 146.6W on this plot. The wind speed cross-section, Figure 7a, shows the jet core is confined within the Arm and the maximum wind speed zone touches the surface which is caused by the incoming southwesterly flow. This air is warmer than the outflow from the Arm which has Interior continental characteristics. It intrudes into the Arm from southeast over the terrain. The interaction can be inferred from the lifting isentropes (Figure 7b) in the low level between 147W and 146.8W.

### 3.3 Simulation of the 7 Mar 2007 case

**Figure 8.**
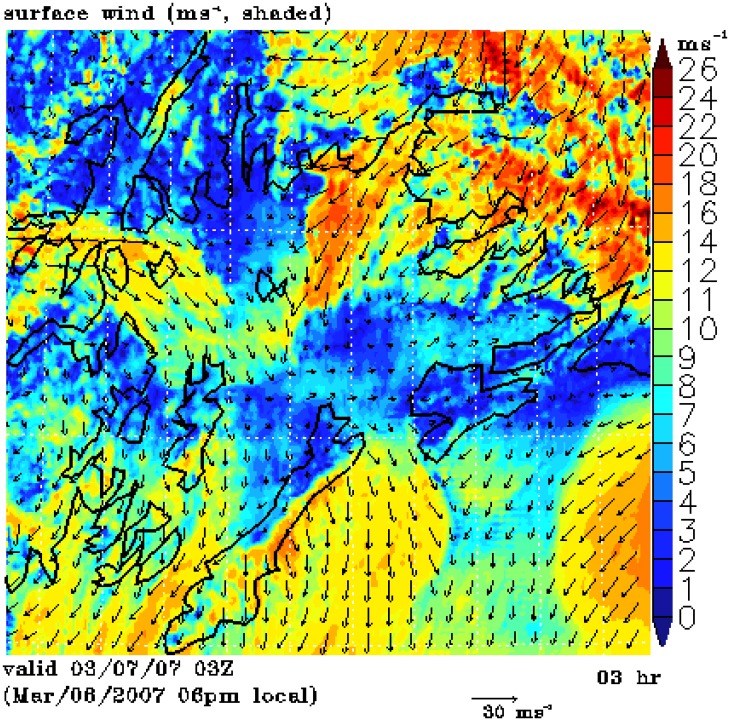
Surface wind for Case B at 3 UTC 7 Mar 2007.

Figure 8 shows the simulation of the 7 Mar 2007 case (Case B), the stronger of the two cases. The simulated Valdez gap wind has a speed of 14 to 20 ms^-1^, the Wells Passage gap wind 12 to 16 ms^-1^. The wind in Port Valdez, which is the northern extension of the Valdez Arm and is west-east trending, is easterly while it is northerly in the adjacent areas and in the Valdez Arm proper. This is all consistent with a strong down-gap flow. The flow in the adjacent areas are north-easterly. As Case A, the air fueling the Valdez Arm gap wind sources from the elevated surrounding terrain, especially from the steep heavily glaciated northern region. The Valdez jet extends more than 20 km southwestwards out of the Arm, where it meets the gap wind traveling eastwards approximately the same distance from the Wells Passage.

**Figure 9.**
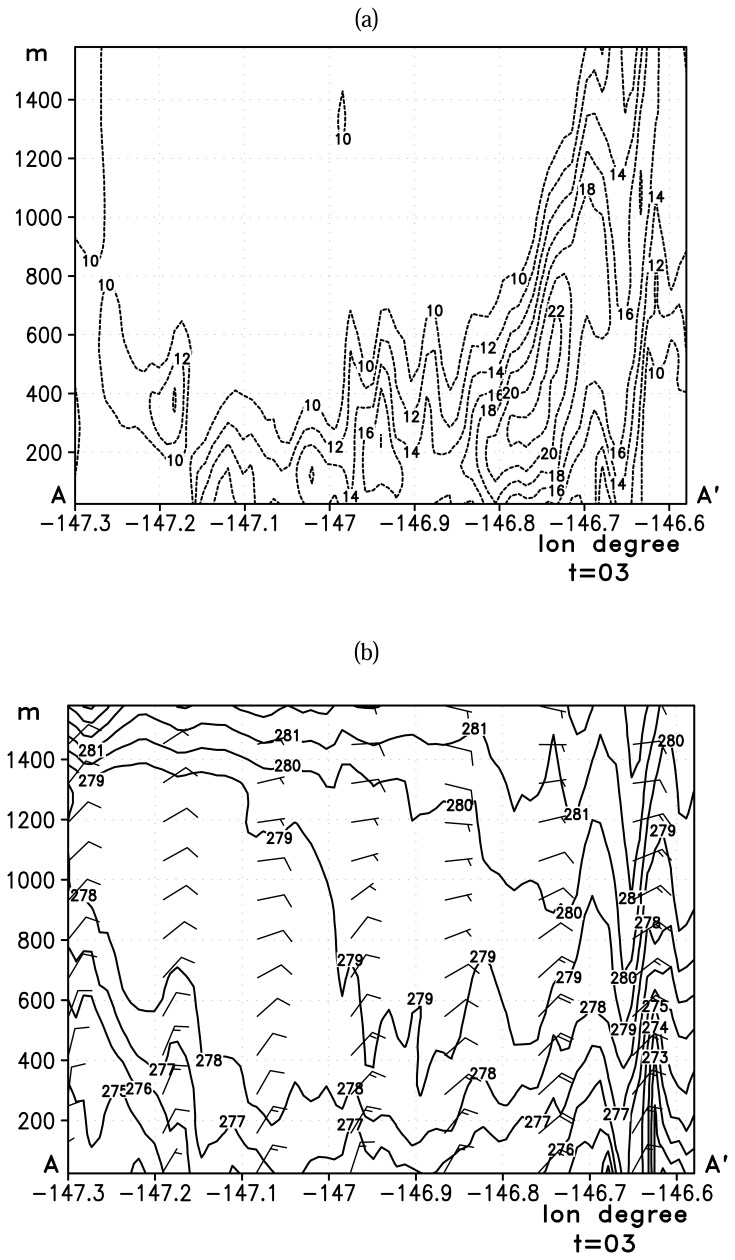
Vertical cross-section along the line AA′ (Figure 5) depicting (a): horizontal wind speed (ms^-1^) and (b): potential temperature (K) along the center of the jet for the Case B. The contour interval is 2 ms^-1^ for the wind speed and 1 K for the potential temperature. The x axis is longitude in degrees and y axis is height above the sea level in meters. A full bar of a wind barb represents 5 ms^-1^.

Figure 9a shows the north-south vertical cross-section of wind for simulated Case B. As with Case A, the maximum speed occurs within the Arm, and the zone of maximum speed coincides with the highest terrain in the two banks. The high speed core terminates right at the exit of the Arm. This abrupt transition in wind speed is due to the change in the terrain [17] such that the jet loses its lateral boundaries. The isentropes (Figure 9b) are descending all the way through the Arm and into the PWS till the jet meets the westerly Wells Passage gap wind. This suggests the air is descending from its “point of origin”— the elevated areas north of the Port Valdez— to the center of the Sound. The depth of the jet lowers significantly once the flow is out of the Arm due to the lack of the constraining forces at its lateral boundaries.

## 4. Discussion

Previous studies showed that gap winds are caused by the pressure gradient along a gap [17, 28] and down slope wind may enhance the flow [8, 2]. Our high-resolution simulations confirm the previous theory but also suggest that other mesoscale processes such as a barrier jet mechanism play an important role in forming and enhancing a gap wind.

### 4.1 Barrier jet enhancement

**Figure 10.**
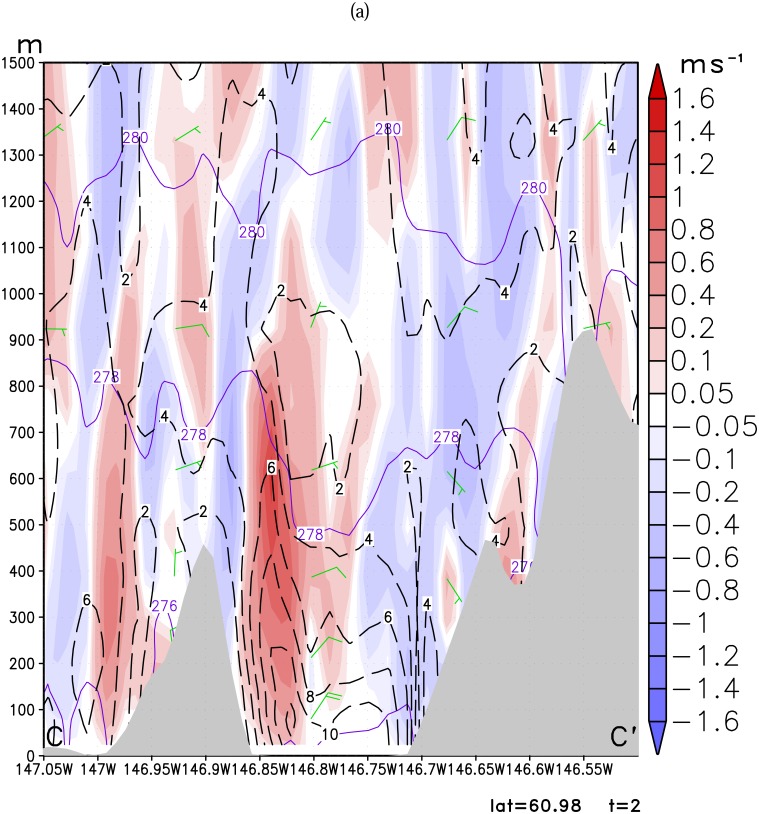

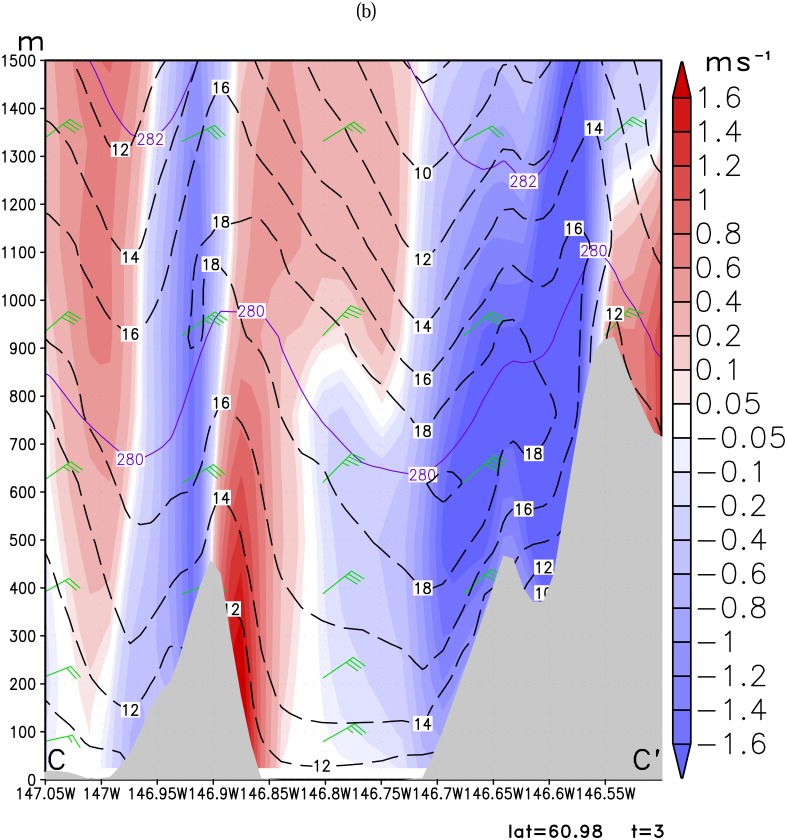
Vertical cross-section of wind speed (ms^-1^, dashed line), potential temperature (K, solid line) and vertical velocity (ms^-1^shaded) along line CC′ (Fig.5) for (a): Case A and (b): Case B. The contour interval of potential temperature is 2 K, wind speed 2 ms^-1^.

Loescher et al [11] found barrier jets tend to exhibit a 2-3 times enhancement of the winds over the ambient synoptic flow. Although it may not be that prominent, we hypothesize that the barrier jet mechanism may play an important role in enhancing a gap wind in the Valdez Arm. This is evidenced by the fact that the simulated Valdez gap wind leans towards a bank of the Arm instead of in the center of the Arm where a typical gap wind core is found. Figure 10 shows the west-east vertical cross-section along the line (CC′ in Figure 5) traversing the middle of the Arm. The Valdez Arm is between 146.85W and 146.7W on this plot. For Case A (Figure 10a), the isentropes are tightly packed indicating that the lower atmosphere is stably stratified. It also shows that air is descending on both east and west sides in the Arm. However, the west side descending motion is only limited to the upper one third of the ridge, adjacent to a strong ascending motion. The ascending motion in the west side of the Arm is caused by the compression of the air in the east side and the blocking of the west bank of the Arm. The east side descending motion dominates the whole eastern part of the Arm.

The streamlines in Figure 12a indicate that flow is moving northwestward into the Arm along the *east* bank of the Arm. These reversing currents compromise the down channel movement of the Valdez Arm gap flow in the east side of the Arm, as a result, the jet core is located in the west side of the Arm instead of the east side, and the wind speed gradients are strong in the area next to the barrier. This is a barrier jet-like feature [19, 5, 25]. The barrier jet enhancement to the Valdez gap wind is also obvious in Case B. The air is sinking in the eastern part of the Arm and the air adjacent to the west bank is rising strongly. The jet core covers the whole Arm but the center (20 ms^-1^) is more close to the east bank. The streamlines in Figure 12b show a wind component normal to the east bank in the upstream of the Arm which suggests the barrier jet component.

### 4.2 Katabatic winds in the Valdez Arm

**Figure 11.**
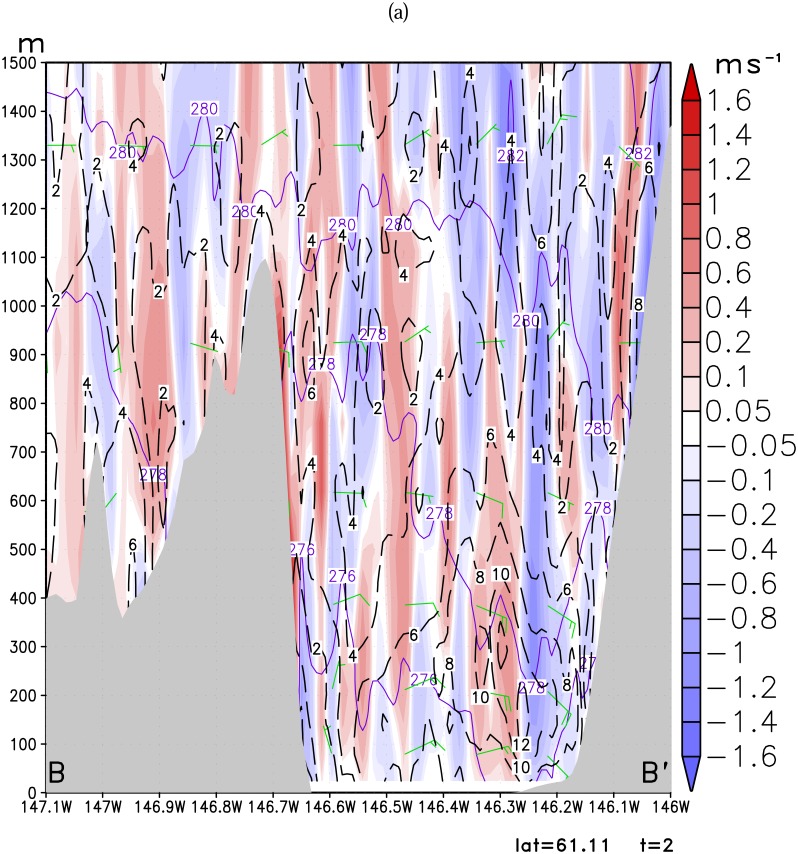

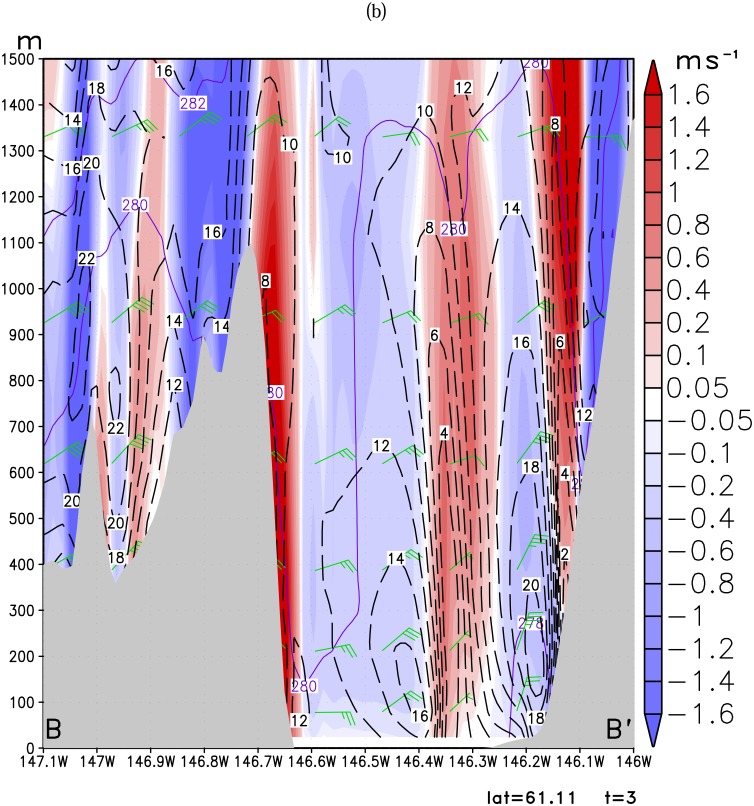
Vertical cross-section of wind speed (ms^-1^, dashed line), potential temperature (K, solid line) and vertical velocity (ms^-1^shaded) along line BB′ (Fig.5) for (a): Case A, and (b): Case B. The contour interval of potential temperature is 2 K, wind speed 2 ms^-1^.

**Figure 12.**
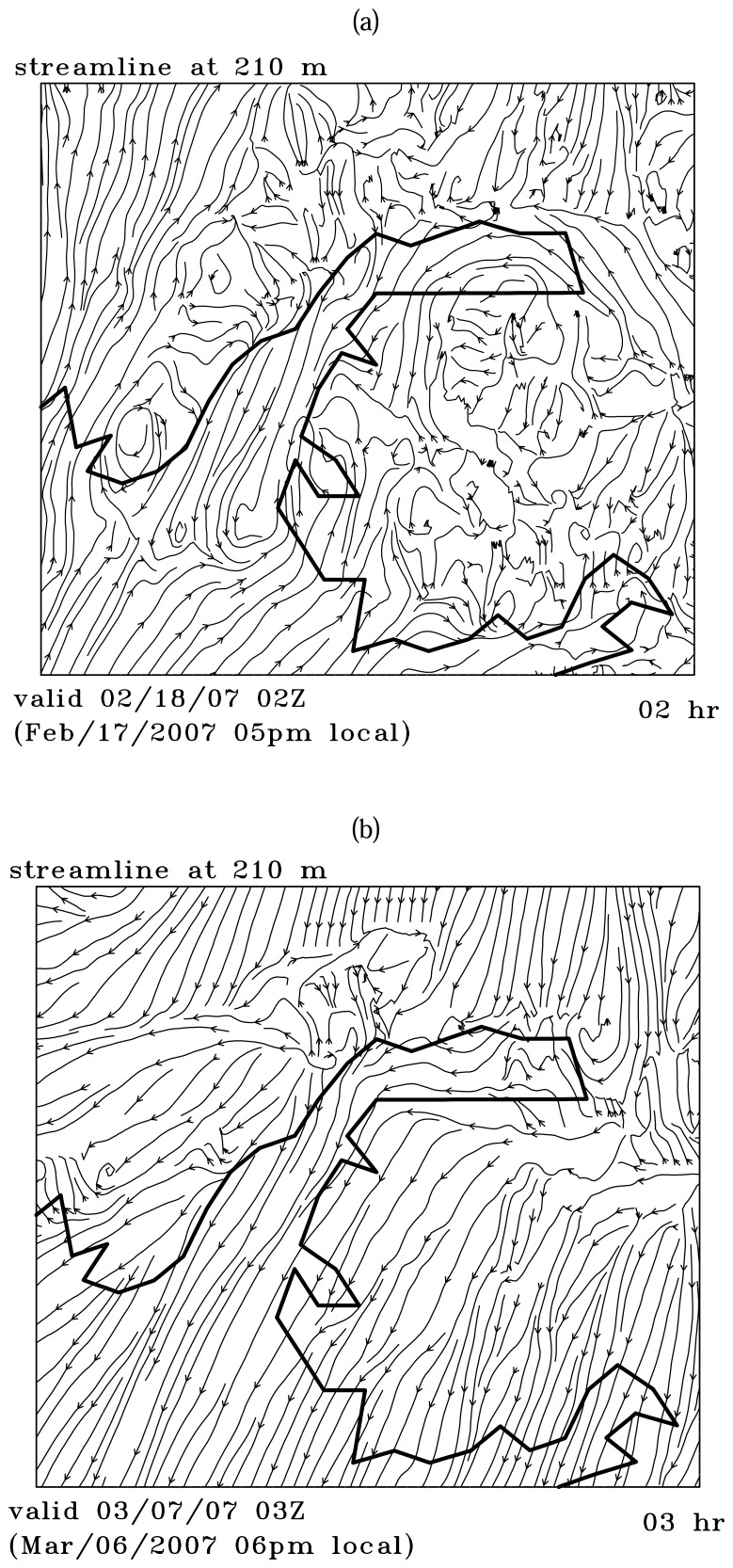
Streamline at 210 m above the ground for (a): Case A and (b): Case B.

The Valdez Arm is surrounded by elevated, often glaciated, terrain from which cold dense air tends to pour into the Arm in the cold season. The two cases studied here illustrate that the leeside winds, blowing into the Arm from surrounding areas, play an important role in fueling the jet in the Arm. Figure 10 shows the west-east cross-section along the parallel 61.11N passing through the Port Valdez (BB′ in Figure 5). The figure shows that winds in the low level are easterly. The air that entered Port Valdez finds its way westwards and turns northerly into the Valdez Arm. The air from the elevated mountain ridges north of the Port drains down into the Port especially through the two valleys of the Mineral Creek and Valdez Glacier (Figure 1). These two drainage flows from Mineral Creek Basin and the Valdez Glacier have a significant interaction with the ambient flow which generates strong upwards movements in the area of contact (Figure 11a and b). Case A has a weak background flow; flows in the west end of the Port split into several more localized streams. Case B has a strong flow; the flow in the west end of the Port is more unified. In the later case, the easterly flow in the Port is so strong that it generates strong upward motion which is caused by the blocking of the west bank of the Arm. Some of the air flows over the barrier and continues westwards to join the ambient flow. The rest of the air mass blocked by the barriers is forced to accelerate along the barrier and down the Arm. The streamline plots (Figure 12a and b), which indicate the flow trajectories, also show the same air flow pattern as stated above.

## 5. Conclusions

High resolution SAR wind imagery and a mesoscale model were combined to better understand gap winds in the PWS. SAR wind imagery reveals detailed wind features over the water body surface while numerical mesoscale model simulations can be used accordingly to understand the highly localized atmospheric interactions over complex coastal terrain. Our focus was on the northeasterly gap wind in the Valdez Arm which is the largest northerly gap in PWS and the origin of the oil tanker route to the continental USA. The SAR wind imagery revealed the gap wind's areal extent and resembled the mesoscale model simulations. The high resolution mesoscale model simulations revealed that the Valdez gap winds were fueled by the down slope winds from the elevated areas surrounding the Arm. Both the weak and strong cases of this gap wind regime showed indications of a barrier jet, stronger winds adjacent to a physical barrier. The west-east vertical cross-sections crossing the middle of the Valdez Arm showed the air is descending on the eastern and rising on the western part of the Arm. The blocking of the barrier induced the upward motion of the air on the western part of the Arm. The eastern bank of the Arm also served as a barrier which accelerated the gap wind in the strong wind case. The westerly winds from the Wells Passage have a significant impact on the Valdez gap wind, especially when the northerly flow is weak. The westerly Wells Passage winds turned southwesterly into the Valdez Arm and suppressed the development of the Valdez gap wind. In an ideal case, pressure gradients are solely responsible for a gap wind, but in “messy” complex coastal areas multiple mesoscale mechanisms likely act in combination to dynamically impact the gap wind. With the addition of SAR-wind imagery, a high-resolution mesoscale model can be used to reveal several of the 3 dimensional details of these interactive flows.

The Alaska SAR demonstration project has found many applications ranging from atmospheric boundary layer measurements, oceanic measurements and sea ice observations [7] — wind retrieval is a part of the atmospheric boundary layer measurements. The SAR wind archive of JHU APL provides a valuable source to begin understanding flows in the extremely complex flow found in PWS. The high spatial resolution of SAR imagery in combination with the mesoscale model's flexible temporal and spatial resolution have proven to be useful in helping elucidate complicated low level flows over complex coastal regions. Reliable availability of SAR snapshots may help improve modeling the atmospheric boundary layer when assimilated into a model. However, this speculation needs to be tested. We anticipate more studies on complicated flows in the Gulf of Alaska using SAR wind retrievals and mesoscale modeling.
